# 6-OHDA-Induced Changes in Colonic Segment Contractility in the Rat Model of Parkinson's Disease

**DOI:** 10.1155/2023/9090524

**Published:** 2023-01-27

**Authors:** Maria Del Pilar Murillo, Ebba Johansson, Victoria Bryntesson, Patrik Aronsson, Gunnar Tobin, Michael Winder, Thomas Carlsson

**Affiliations:** Department of Pharmacology, Institute of Neuroscience and Physiology, The Sahlgrenska Academy, University of Gothenburg, Box 431, Gothenburg SE-40530, Sweden

## Abstract

**Background:**

Gastrointestinal dysfunction is one of the most common non-motor symptoms in Parkinson's disease (PD). The exact mechanisms behind these symptoms are not clearly understood. Studies in the well-established 6-hydroxydopamine (6-OHDA) lesioned rats of PD have shown altered contractility in isolated circular and longitudinal smooth muscle strips of distal colon. Contractile changes in proximal colon and distal ileum are nevertheless poorly studied. Moreover, segments may serve as better tissue preparations to understand the interplay between circular and longitudinal smooth muscle. This study aimed to compare changes in contractility between isolated full-thickness distal colon muscle strips and segments, and extend the investigation to proximal colon and distal ileum in the 6-OHDA rat model.

**Methods:**

Spontaneous contractions and contractions induced by electrical field stimulation (EFS) and by the non-selective muscarinic agonist methacholine were investigated in strip and/or segment preparations of smooth muscle tissue from distal and proximal colon and distal ileum in an *in vitro* organ bath comparing 6-OHDA-lesioned rats with Sham-operated animals. *Key Results*. Our data showed increased contractility evoked by EFS and methacholine in segments, but not in circular and longitudinal tissue strips of distal colon after central 6-OHDA-induced dopamine denervation. Changes in proximal colon segments were also displayed in high K^+^ Krebs-induced contractility and spontaneous contractions.

**Conclusions:**

This study further confirms changes in smooth muscle contractility in distal colon and to some extent in proximal colon, but not in distal ileum in the 6-OHDA rat model of PD. However, the changes depended on tissue preparation.

## 1. Introduction

One of the most common non-motor symptoms that significantly affect the quality of life of patients with Parkinson's disease (PD) is gastrointestinal dysfunction, which includes constipation, diarrhea, and fecal incontinence (for reviews see References [1, 2]). Clinical studies have shown that more than half of all PD patients report decreased frequency of defecation and problems with stool expulsions, which is a significantly higher ratio as compared with healthy subjects [3, 4]. The gastrointestinal symptoms have also been shown to worsen as the disease progresses, which is more common in later Hoehn and Yahr stages of PD [4]. However, no disease-specific drug treatment options for the gastrointestinal symptoms are available in PD patients [5]. The gold standard PD drug treatment L-DOPA has even been suggested to potentially worsen the gastrointestinal function by inhibiting the gastric emptying and affecting its own drug absorption patterns in healthy subjects [6]. Interestingly, gastrointestinal symptoms are known to often develop very early in the disease, even before the characteristic motor symptoms, and thus the diagnosis of the disease. This renders these symptoms of particular interest regarding the identification of possible biomarkers of PD [4, 7].

The mechanisms behind these troublesome non-motor symptoms in PD are poorly understood. The disorder is known to affect both the enteric nervous system as well as the central nervous system [2], which may both be responsible for the development of the gastrointestinal problems. To elucidate the mechanisms, most studies have used the 6-hydroxydopamine (6-OHDA) rat model of PD [8–22]. In this model, selective central dopamine (DA) neuron degeneration is induced, which in fact shows signs of PD-like gastrointestinal dysfunction [1, 23]. Specifically, in isolated colon muscle preparations, alterations in motility, represented by increased peristaltic waves and decrease wave amplitude, have been observed [12, 18]. Furthermore, significant changes in response to electrical stimulation and cholinergic receptor activation in isolated longitudinal and circular smooth muscle preparations of distal colon have also been demonstrated in the same 6-OHDA model [11, 13, 14]. Although previous studies have investigated the contractile responses of the distal colon, we propose that an intact tissue segment provides more comprehensive information regarding the physiological changes occurring locally in the intestine when investigated *in vitro*. Specifically, we would argue that full-thickness segments include more complex connections with complete mucosal structures and connections between the different layers of the smooth muscle.

In the current study, we aimed to investigate distal colon contractility using tissue segments and compare this to the more commonly used circular and longitudinal muscle strips, in an *in vitro* organ bath setup in the 6-OHDA rat model of PD. Furthermore, we aimed to explore possible contractile alterations also in the proximal colon and distal ileum. In addition, in an aim to ethically refine the protocols when studying smooth muscle contractility in the 6-OHDA rat model, we also included normal, healthy, animals to investigate if untreated (with non-invasive surgery) animals can replace the Sham-operated control animals.

## 2. Materials and Methods

### 2.1. Animals and Ethics

A total of 50 adult male Sprague–Dawley rats (Charles-River SRL, Calco, Italy), age 8–16 weeks and weighing 260–560 g at the beginning of the experiments, were housed under standard laboratory conditions on a 12 hours/12 hours light/dark cycle. Animals had access to food and water *ad libitum*, and acclimatized for one week before starting the experiments. The study was approved by the local ethical committee (no. 145/15, date approved: October 2, 2015, and 1911/21, date approved: January 27, 2021). All procedures and animal care were carefully considered to minimize the pain and discomfort of the animals included in the study, and which followed the Directive 2010/63/EU of the European Parliament and of the Council of the European Union (Document 32010L0063).

### 2.2. Experimental Design

The animals were first divided into two groups, which received brain surgery, either by an injection of the 6-OHDA neurotoxin (6-OHDA; *n* = 20), or as control by an injection of saline (Sham, *n* = 20) into the nigrostriatal pathway. Four weeks (25–31 days) post-lesion, the 6-OHDA- and Sham-lesioned animals were sacrificed and tissue samples of the distal and proximal colon, and the distal ileum, were collected for *in vitro* analysis of smooth muscle function in both circular and longitudinal tissue strips and tissue segments. The brains were fixed by transcardial perfusion with 4% paraformaldehyde (PFA), sectioned, and immunohistochemically stained against dopaminergic neurons using tyrosine hydroxylase (TH) antibodies. In addition to the lesioned animals, untreated rats (Healthy, *n* = 10) were added to further evaluate smooth muscle functions as compared with the Sham-operated controls. The muscle contractility of all animals and tissue samples were evaluated in an isolated organ bath setup using electrical field stimulation (EFS) and direct cholinergic receptor stimulation by the non-selective muscarinic agonist methacholine. In the proximal colon and distal ileum, the EFS- and methacholine-induced contractility was further evaluated in the presence of the cholinergic antagonist atropine. In the distal colon, a subset of animals was evaluated after atropine, whereas a second subset was evaluated in the presence of the nitric oxide (NO) synthase inhibitor L-NAME. Additional segments from the latter animals were inverted inside-out, that is, exposing the luminal space (in contrast to the regular segments, where the luminal space was closed) and evaluated for EFS and methacholine-induced contractility in absence and presence of L-NAME. Spontaneous contractions were finally analyzed in all tissues. The experimental design and timeline are shown in Supplemental Figure 1.

The viability of the intestinal tissue was evaluated at start of the *in vitro* experiment by application of high-potassium (124 mM) Krebs solution (“high K^+^ Krebs”). This revealed unviable responses (<7 mN in high K^+^ Krebs response, including absence of both EFS and methacholine responses) in some of the tissue preparations. The EFS data further revealed lack of frequency-dependent responses in a set of the viable tissues. These data, which were evenly distributed through both different groups and respective tissue preparations, were thus excluded from the data analysis. The number of included tissue samples in the Healthy, Sham, and 6-OHDA groups for EFS and methacholine are indicated in Table 1.

### 2.3. Nigrostriatal 6-OHDA Lesion

Unilateral DA- or Sham lesions were performed by injection of 6-OHDA or saline, respectively, in the right medial forebrain bundle of the brains, as previously described [24]. In brief, the animals were placed in a stereotaxic frame (Kopf Instruments, Tujunga, CA, USA) under 1.5–2.5% isoflurane (in air; Forene Abbott, Wiesbaden, Germany) general anesthesia maintaining body temperature at 38°C by a heating pad. Local anesthesia (Marcain 2.5 mg ml^−1^, AstraZeneca AB, Stockholm, Sweden) was applied before a sagittal incision of ≈1 cm was made on the scalp. A burr hole was further drilled at coordinates, according to Paxinos and Watson [25], and to bregma as follows: anterior–posterior: −4.4 mm, medial–lateral: −1.2 mm, and dorsal–ventral: −7.8 mm; with the toothbar set at −2.4 mm. The animals received 4 *μ*l of either 6-OHDA (3.5 *μ*g *μ*l^−1^ dissolved in saline solution containing 0.02% of ascorbic acid; Sigma–Aldrich, St. Louis, MO, USA) or sterile saline solution at the injection speed of 1 *μ*l min^−1^ using a NanoFil 10 *μ*l syringe coupling to a 33-gauge blunt needle (World Precision Instruments, Inc., Sarasota, FL, USA). The needle was then kept in place for 3 minutes, and then slowly retracted. At the end of the procedure, the skin wound was closed with suture clips and the animals received post-analgesia (Romefen, 5 mg kg^−1^ subcutaneously; VET, Merial, Lyon, France) during recovery.

### 2.4. Tissue Extraction and Brain Fixation

**Figure 1:**
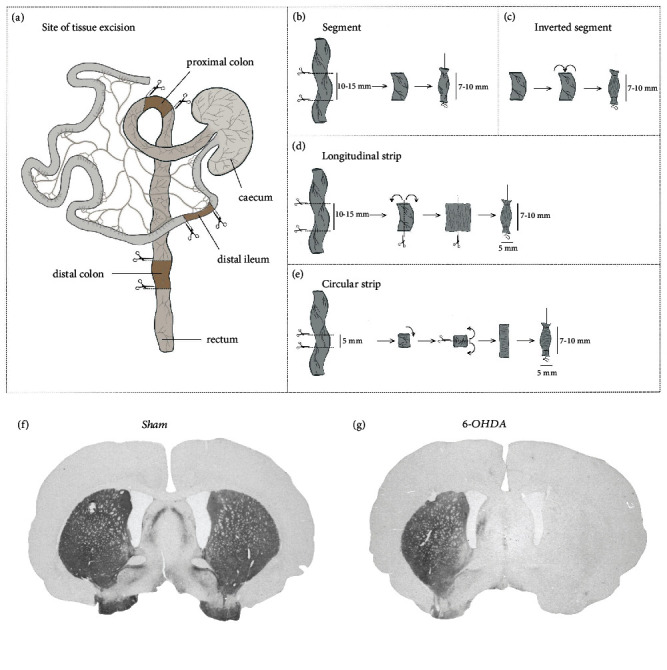
Tissue dissection and preparation and 6-OHDA-induced dopamine (DA) lesion. The distal colon, proximal colon, and distal ileum tissues were excised by first cutting three, each 4–5 cm long tissue pieces, from ≈3 cm from the anus, and 3 cm from each side of the aboral and oral end of the caecum, respectively (shaded sections in a). Segments were then prepared from these 4–5 cm long tissue pieces by further cutting ≈10–15 mm long tubes of each tissue type (scissor markings), followed by attaching a silk thread to each end closing the luminal space (b). In a subset of the ≈10–15 mm long tubes, inverted segments were created by turning the tissue inside out, exposing the luminal space but closing the mucosal space (c). The longitudinal and circular strips were also prepared from the 4 to 5 cm long tissue pieces. The longitudinal strip was prepared by cutting a ≈10–15 mm long segment, opening it up along the mesenteric line, splitting in half with a width of ≈5 mm, and attaching a thread at each end (d). The circular strips were prepared by cutting a ≈5 mm long segment, rotating it 90°, opening it up along the mesenteric line, and attaching a thread at each end (e). The saline injection (Sham) did not affect the DA fibers (f), whereas the 6-OHDA injections (6-OHDA) in the medial forebrain bundle induced an almost complete DA fiber degeneration in the striatum (g).

The 6-OHDA and Sham animals, at 25−31 days post-injection, and the Healthy animals were sacrificed with an overdose of pentobarbital sodium (100–150 mg kg^−1^, intraperitoneal injection, APL, Stockholm, Sweden). Just before cardiac arrest, an abdominal midline incision was made and three intestinal segments, each 4–5 cm long, were excised [shaded sections between scissors in Figure 1(a)]. These were collected ≈3 cm from the rectum (distal colon), and 3 cm on each side of the caecum, that is, from the aboral end (proximal colon) and from the oral end (distal ileum). The tissues were further transported in room-temperature Krebs solution [NaCl, 118  mM; KCl, 4.6  mM; KH_2_PO_4_, 1.15  mM; MgSO_4_ (anhydrous), 1.15 mM; NaHCO_3_, 25  mM; CaCl_2_, 1.25 mM; and C_6_H_12_O_6_ (glucose), 5.5 mM] and segments and circular and longitudinal strips were prepared within one hour for functional experiments in the organ bath [Figures 1(b), 1(c), 1(d), and 1(e)]. Following intestinal tissue extraction, the animals were transcardially perfused with 4% PFA [in 0.1 M phosphate buffer (PB); Sigma–Aldrich] as described previously [26]. In brief, through a needle in the animals right ventricle, about 30 ml 0.9% NaCl (at room temperature) followed by 200–250 ml ice-cold 4% PFA (in 0.1 M PB) was infused over 8–10 minutes, and the brains were removed and post-fixed in PFA overnight. They were then transferred to 25% sucrose solution (in 0.1 M PB) for at least 24 hours before cutting the brains in 35 *μ*m slices (in five series) with a cryotome (Leica, CM1950; Leica Microsystems, Nussloch, Germany). The sections were stored in cryoprotectant (25% ethylene glycol + 25% glycerol in 0.1 MPB) until further immunohistochemistry staining.

### 2.5. Tissue Preparations and Organ Bath Experiments

The colon and ileum tissue segments were prepared as previously described [27]. Briefly, the luminal contents were first gently flushed out from the three collected, each 4–5 cm long, intestinal tissues [distal and proximal colon and distal ileum; shaded sections between scissors in Figure 1(a)] using room-temperature Krebs solution. From each of these three intestinal tissues one segment, ≈10–15 mm long, was cut and the ends were tied using silk thread (Vömel, Kronberg, Germany), closing the luminal space [scissor markings in Figure 1(b)]. An additional set of the 10–15 mm long segments from the distal colon was further gently turned inside out where each end was tied exposing the luminal space [Figure 1(c)]. From the 4 to 5 cm long distal and proximal colon tissues, longitudinal and circular tissue strips were also prepared as described in Figures 1(d) and 1(e). Briefly, the longitudinal colon strip was prepared by first cutting ≈10–15 mm of a segment followed by a cut at the mesentery line exposing the internal part of the tissue. It was further opened and cut in half, at a width of ≈5 mm in the vertical direction and tied at each end of the tissue using silk thread ≈7–10 mm apart [Figure 1(d)]. The circular strip was prepared by first cutting ≈5 mm of a colon segment followed by a cut at the mesentery line exposing the internal part of the tissue. It was then rotated 90° and tied at both ends ≈7–10 mm apart using silk thread [Figure 1(e)[. All tissue segments and tissue strips were then mounted to an organ bath setup (Linton Instrumentation, Norfolk, UK or BIOPAC Systems, Inc., Goleta, CA, USA).

The isolated tissue baths contained 20 or 25 mL of Krebs solution aerated with a mixture of 95% O_2_ and 5% CO_2_ at 37°C. Intestinal contractility was recorded using a calibrated isometric force transducer (TSD125C or SS63L, BIOPAC systems, Inc.). First, the tissues following mounting were left to equilibrate for 45 minutes at a basal tension of 10–15 mN. The tissue was then challenged with high K^+^ Krebs (124 mN replacing equimolar amount of Na^+^ for K^+^ in the solution) three times to test the viability and activate the tissues. After a washout period of at least 10 minutes and after the tension had returned to baseline, the tissue was challenged with EFS at 1, 2, 5, 10, 20, and 40 Hz (at supra-maximal voltage; delivered as square wave pulses with a duration of 0.8 ms, until the peak response was obtained). This was followed by a challenge with cumulative concentrations of the cholinergic muscarinic agonist, methacholine (10^−8^–10^−3^ M; Sigma–Aldrich). The tissues were further once again challenged with EFS and methacholine at 20 minutes after addition of either the non-selective cholinergic antagonist, atropine (10^−6^ M) or, for a subset of distal colon segments, the NO synthase inhibitor L-NAME (10^−6^ M). Washout periods of at least 10 minutes were applied between each set. Finally, a second stimulation with high K^+^ Krebs solution was performed in each tissue to test the viability at the end of the experiment. The tissue segments and tissue strips were lastly removed from the organ bath, briefly dried, and the wet-weight was measured.

#### 2.5.1. Acquisition and Data Analysis

The contractile responses were recorded and analyzed using the MP100WSW data acquisition system with the Acknowledge Software v4.3 or BSL student v4.1 (BIOPAC systems, Inc.). The EFS was analyzed by subtracting the mean basal tension, over 10–30 seconds prior to stimulation for each frequency, from the maximum peak value. The response to methacholine was calculated by subtracting the mean peak value for any given concentration and the mean basal tension, over 10–30 seconds, prior to administration of the first concentration. The EFS and methacholine responses were finally calculated as contraction per mg tissue (mN mg^−1^) by dividing the absolute contraction with the wet-tissue weight.

The frequency of the spontaneous contractions was evaluated over 5 minutes after the high K^+^ Krebs activation, but just prior to the first EFS challenge. Each peak greater than 1 mN was counted as a peak and the respective amplitude was measured, where the average amplitude for each animal was computed.

### 2.6. TH-Immunohistochemistry

To identify the DA fibers in the striatum, free-floating sections were immunohistochemically stained against the TH-enzyme as previously described [24]. In brief, the sections were first quenched using 3% H_2_O_2_ (Sigma–Aldrich) and 10% methanol in phosphate-buffered saline (PBS), and second pre-incubated for one hour in 5% normal horse serum (Vector Laboratories, Burlingame, CA, USA) and 0.25% Triton-X in PBS. This was followed by an overnight incubation with a mouse anti-TH antibody (1 : 1000, MAB318, Merck Millipore, Billerica, MA, USA and/or 1 : 1000, #22941, Immunostar, Inc. Hudson, WI, USA) in the same solution. Following rinses, the sections were incubated for one hour in biotinylated horse anti-mouse (1 : 250, BA2001, Vector Laboratories), followed by one-hour incubation in avidin–biotin complex solution (ABC Elite, Vector Laboratories). Finally, the staining was revealed by adding 3,3′-diaminobenzidine (Vector Laboratories) and 0.01% H_2_O_2_. The sections were mounted on glass slides (HistoBond+, Marienfeld, Lauda-Königshofen, Germany) and coverslipped using Depex mounting agent (Merck KGaA, Darmstadt, Germany). All steps were performed at room temperature with samples kept under continuous motion on a shaking plate (KS125 Basic, IKA, Staufen, Germany), and between each step the sections were washed 3 × 10 minutes with PBS.

#### 2.6.1. Striatal TH-Positive Fiber Density Measurements

To estimate the extent of DA lesion in the striatum, the mean optical density at six striatal levels, according to Paxinos and Watson [25], corresponding to +2.16, +1.56, +0.96, +0.36, −0.36, and −0.96  mm from the bregma, was measured using the ImageJ bundled with 64-bit Java v. 1.8.0 (NIH, Bethesda, MD USA; http://imagej.nih.gov/ij/). The striatum was outlined as previously described by Carlsson et al. [28]. Briefly, the included area followed the lateral ventricle, the corpus callosum, the external capsule, and the anterior commissure. The corpus callosum in respective ipsilateral hemisphere was used as background staining. The images were captured by the Dino-Lite digital microscope system (Dino-Lite Edge AM4515T8, AnMo Electronics Cooperation, Taipei, Taiwan) and the DinoCapture 2.0 software.

### 2.7. Statistical Analysis

**Figure 2:**
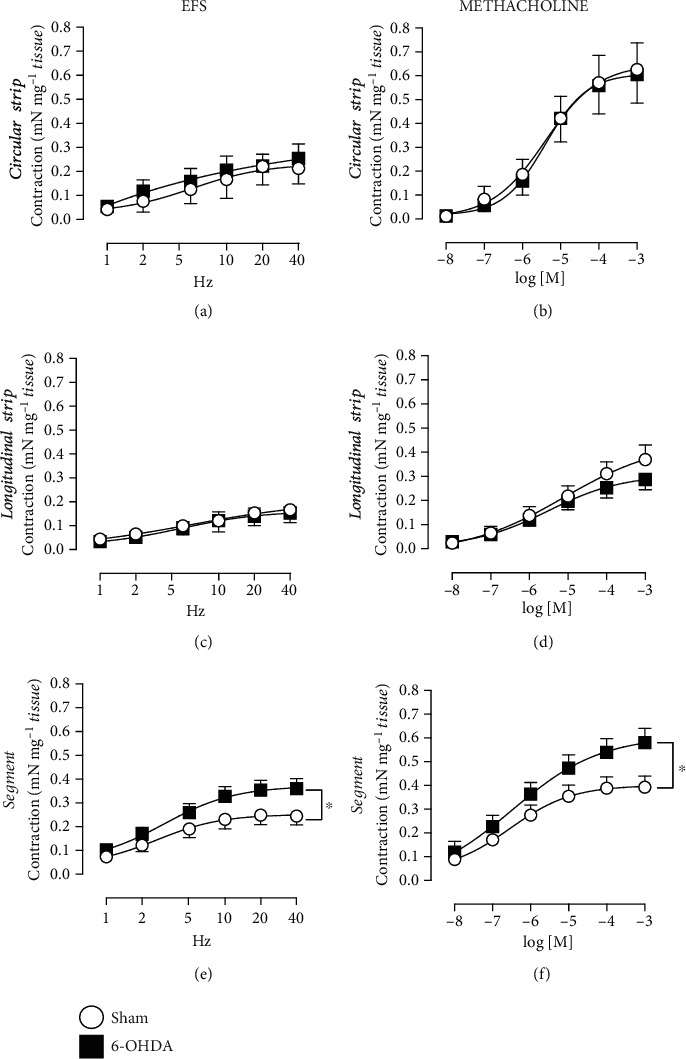
Distal colon contractile responses to electrical field stimulation (EFS) and methacholine. Following EFS, frequency-dependent responses were observed in all tissues. The segments showed significant differences in the 6-OHDA group in both EFS- (e) and methacholine-induced contractions (f) compared with Sham (*n* = 16 and *n* = 17, respectively). However, no differences in EFS- and methacholine-mediated contraction were observed between the 6-OHDA and Sham animals in circular (a and b; *n* = 10 and *n* = 9, respectively) and longitudinal strips (c and d; *n* = 9 and *n* = 8, respectively). ∗ = Significant difference from Sham group; two-way ANOVAs (e) group-concentration interaction (5, 155) = 3.88, *p* = 0.0024; (f) group-concentration interaction (5, 155) = 4.38, *p* = 0.0009.

**Figure 3:**
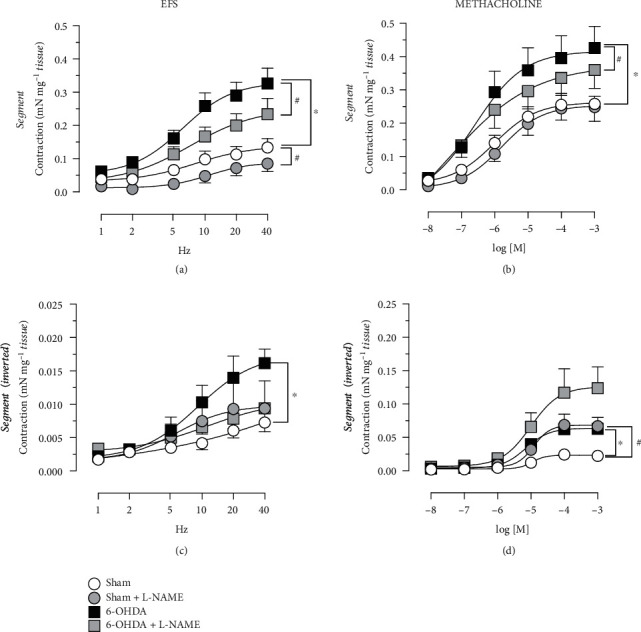
Contractions of distal colon segments and inverted segments following electrical field stimulation (EFS) and methacholine in the absence and presence of the nitric oxide synthase inhibitor L-NAME. Significant and similar reduced contractile responses in EFS by L-NAME were observed in the segments in both Sham (*n* = 6) and 6-OHDA animals (*n* = 5; a). However, L-NAME reduced the methacholine-mediated contractility in the 6-OHDA animals (*n* = 6), but not in the Sham group (*n* = 5; b). The inverted segments showed significant increased responses to EFS (c) and methacholine (d) in the 6-OHDA (*n* = 4) as compared with Sham group (*n* = 6) in the absence of L-NAME. In the presence of L-NAME the EFS response showed no difference within respective groups, but a trend to decrease could be seen in the 6-OHDA animals. The methacholine response on the other side was significantly increased in the Sham group after L-NAME. The increase was however, not significant in the 6-OHDA animals. ∗Significant difference from Sham group; # = significant difference between presence and absence of L-NAME in respective group. Two-way ANOVAs (a) ∗ = group *F*(1, 9) = 15.31, *p* = 0.00036; and # = group *F*(1, 5) = 14.29, *p* = 0.0129; *F*(1, 4) = 8.44, *p* = 0.0438 for Sham and 6-OHDA, respectively; (b) ∗ = group *F*(1, 9) = 6.36, *p* = 0.0327 and # = group *F*(1, 4) = 7.84, *p* = 0.0488 for 6-OHDA; (c): ∗ = group *F*(1, 8) = 6.80, *p* = 0.0312; (d) ∗ = group *F*(1, 8) = 38.32, *p* = 0.0003; # = *F*(1, 5) = 18.72, *p* = 0.0075 for Sham. The data here are representing a subset of the data included in Figures 2(e) and 2(f).

High K^+^ Krebs, EFS, and methacholine contractility data are presented as mean ± standard error of the mean (SEM) of the tissue weight-corrected contractions (mN mg^−1^), or as median with individual data points for the spontaneous contraction data. Statistical analysis and graphs were performed using the GraphPad Prism 9.1.1 for Mac OS (GraphPad Software, Inc., San Diego, CA, USA). The group- and group-concentration interaction comparisons were done using student's unpaired *t*-test or two-way Analysis of variance (ANOVA) with Sidak *post-hoc* test or non-parametric Mann–Whitney *U* test where appropriate. A *p*-value less than 0.05 was considered statistically significant. Statistics for significant changes and non-significant trends are presented in respective figure legend. A subset of the data from Figures 2(e) and 2(f) are also represented in Figures 3(a) and 3(b).

## 3. Results

### 3.1. Dopamine Degeneration

The TH-immunohistochemistry of the striatum revealed that four animals in the 6-OHDA group showed incomplete DA lesion and two in the Sham-operated group displayed a partial DA loss and were therefore excluded from the study. The DA fiber density measurement for the included animals showed an almost complete DA denervation in the 6-OHDA group (3.8 ± 0.4% of intact side, *n* = 16; Figure 1(g)), whereas the Sham animals remained intact (97.2 ± 1.0% of intact side, *n* = 18; unpaired *t*-test, *p* < 0.0001; Figure 1(f)). Further visual evaluation of the DA neurons in the substantia nigra showed, as expected, also an almost complete degeneration as previously reported [29].

### 3.2. Rat and Tissue Weights

The rat body weight at sacrifice was similar in the Sham and 6-OHDA groups (500 ± 18 g, *n* = 18 and 488 ± 14 g, *n* = 16, respectively, *p* = 0.628). The weight of the circular and longitudinal distal and proximal colon tissue strips as well as the distal colon, proximal colon, and distal ileum segments also showed no significant differences between the two groups (Table 2). However, trends of smaller ileum segments and larger proximal colon circular strips in the 6-OHDA animals could be observed (Table 2). Comparing the Sham animals with Healthy animals showed no difference between the groups in either rat weight or circular and longitudinal strips or segments weight, although, a trend towards larger distal colon segments was observed in the Sham animals (Table 2).

### 3.3. Distal Colon

The high K^+^ Krebs responses in the Sham and the 6-OHDA groups were similar in both circular and longitudinal strip preparations (Table 2). The response of the distal colon segments was however, significantly increased by 65%, from 0.31 ± 0.034 mN mg^−1^ tissue in the Sham animals to 0.51 ± 0.046 mN mg^−1^ tissue in the 6-OHDA animals (Table 2).

Following EFS and methacholine no significant differences in contractility (mN mg^−1^) were observed in the circular [Figures 2(a) and 2(b)] or longitudinal strips [Figures 2(c) and 2(d)] between the 6-OHDA and Sham animals. Similar as the high K^+^ Krebs response, a significant increased contractile response to both EFS [Figure 2(e)] and to methacholine [Figure 2(f)] was revealed in the segments of the 6-OHDA group. This reached about 50% increase in both EFS and methacholine at the highest given frequency and concentration, respectively. Following atropine, significant inhibition of the contractions from EFS and methacholine were observed in all tissues and both groups, but no differences were observed between the groups. The EFS response, at 10 Hz, reached 70–100% inhibition, and the methacholine-induced contraction, at 10^−5^ M, reached 71–99% inhibition, in the different tissues and groups in the presence of atropine.

In the subset of the segments where the effects of L-NAME were also investigated, significant, 36% and 51% decreased EFS responses in both Sham and 6-OHDA animals were observed, respectively [Figure 3(a)]. Interestingly, the methacholine response decreased significantly in the 6-OHDA, by an 18% inhibition at 10^−5^ M, but not in the Sham group following the L-NAME treatment [Figure 3(b)].

The subset of distal colon tissue where the tissue was turned inside out, hence exposing the mucosa (segment inverted) showed, similar as segments, significantly higher EFS- [Figure 3(c)] and methacholine-induced [Figure 3(d)] responses in the 6-OHDA group as compared with Sham animals. Following treatment with the NO synthase inhibitor L-NAME (10^−6^ M), the EFS showed no significant change in either of the groups [Figure 3(c)]. The effects of L-NAME on the methacholine-induced contractility showed increased responses; however, only reaching significance in the Sham group [Figure 3(d)].

### 3.4. Proximal Colon

In the proximal colon segments a 43% significant increase in contraction following high K^+^ Krebs was observed (Sham: 0.30 ± 0.026 mN mg^−1^ tissue vs. 6-OHDA: 0.43 ± 0.031 mN mg^−1^ tissue; Table 2). For the circular and longitudinal strip tissues no changes in high K^+^ Krebs evoked responses between the two groups could be observed (Table 2).

**Figure 4:**
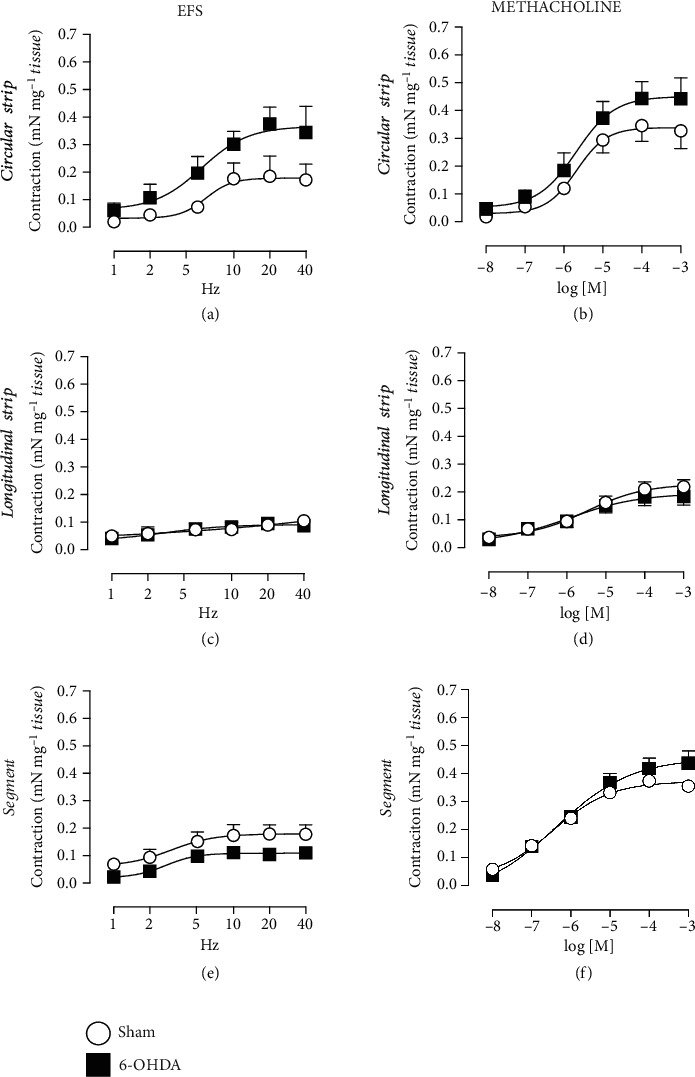
Proximal colon contractile responses to electrical field stimulation (EFS) and methacholine. EFS in the circular strips showed a trend for increased contractile responses in the 6-OHDA (*n* = 7) as compared with Sham animals (*n* = 6; a), whereas the proximal colon segments displayed a non-significant decreased response in the 6-OHDA animals (*n* = 9) compared with Sham animals (*n* = 8; e). The methacholine-induced contraction in the circular strips (b: 6-OHDA, *n* = 10; Sham, *n* = 7) and the segments (f: 6-OHDA, *n* = 11; Sham, *n* = 11) was not significantly changed between the groups. No changes were observed in the longitudinal strips either by EFS (c; 6-OHDA, *n* = 7 and Sham, *n* = 6) or methacholine-induced contraction (d; *n* = 10 in each group); two-way ANOVAs a: group *F*(1, 11) = 3.30, *p* = 0.0966; (e) group *F*(1, 15) = 3.41, *p* = 0.0848.

The 6-OHDA animals showed a trend to increase in tissue weight-corrected response in the circular strips following EFS [Sham: 0.19 ± 0.073 mN mg^−1^ tissue, 6-OHDA: 0.38 ± 0.061 mN mg^−1^ tissue at 20 Hz; Figure 4(a)]. The EFS response in the longitudinal strips showed no difference between the 6-OHDA and the Sham animals [Figure 4(c)]. The EFS response in the segment of the proximal colon showed a non-significant decrease in the 6-OHDA group, as compared with Sham animals, where the segments contracted, at 20 Hz, 0.18 ± 0.034 mN mg^−1^ tissue in the Sham group and 0.10 ± 0.021 mN mg^−1^ in the 6-OHDA animals [Figure 4(e)]. Following methacholine-induced contraction, none of the circular [Figure 4(b)], longitudinal strips [Figure 4(d)], or segments [Figure 4(f)] displayed any significant differences in contractility in the 6-OHDA animals as compared with the Sham group. The circular nevertheless visually displayed an increase in smooth muscle contractility at the higher concentrations of methacholine in the 6-OHDA group [at 10^−3^ M Sham: 0.33 ± 0.064 mN mg^−1^ tissue vs. 6-OHDA: 0.44 ± 0.075 mN mg^−1^ tissue; Figure 4(b)]. In the presence of atropine (10^−6^ M), the average inhibition of the EFS response reached, at 20 Hz, 61–103% and for the methacholine, at 10^−5^ M, 63–97%, for all tissues in the Sham and 6-OHDA groups.

### 3.5. Distal Ileum

**Figure 5:**
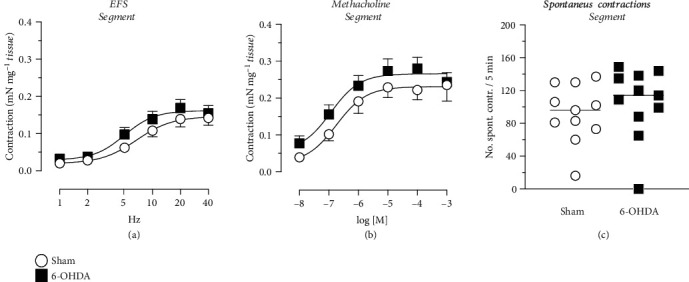
Electrical field stimulation (EFS) and methacholine-induced contractions in distal ileum segments. No significant difference in the smooth muscle contractility (mN mg^−1^) was observed after either EFS (a: Sham *n* = 10; 6-OHDA, *n* = 9) or methacholine (b: Sham *n* = 11; 6-OHDA, *n* = 11). In addition, the frequency of the spontaneous contraction was unaltered by 6-OHDA-induced dopamine lesions (c: Sham *n* = 11; 6-OHDA, *n* = 11).

No changes in high K^+^ Krebs response were observed in the distal ileum segments between the Sham and the 6-OHDA groups (Table 2). Furthermore, no changes in weight-corrected responses to EFS [Figure 5(a)] or methacholine [Figure 5(b)] were observed in the 6-OHDA group as compared with the Sham animals. In the presence of atropine similar inhibition of both EFS (at 10 Hz, 76% and 65% in the Sham and 6-OHDA group, respectively) and methacholine (at 10^−5^ M, 71% and 61% in the Sham and 6-OHDA group, respectively) contractions were observed in the two groups.

### 3.6. Spontaneous Contractility

**Figure 6:**
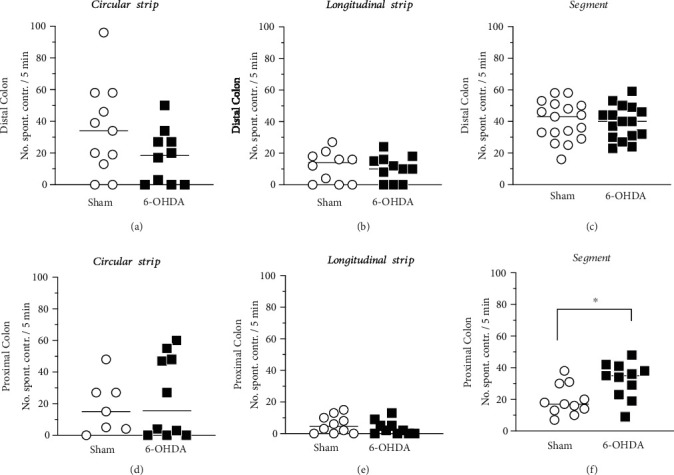
Spontaneous contractions in distal and proximal colon. No changes were observed between the groups in distal colon in either circular strips (a: Sham *n* = 10; 6-OHDA, *n* = 11), longitudinal strips (b: Sham *n* = 10; 6-OHDA, *n* = 11) or segments (c: Sham *n* = 17 6-OHDA, *n* = 16). In the proximal colon segments (f) an increased spontaneous contractility after the high K^+^ Krebs activation were observed in the 6-OHDA (*n* = 11) as compared with Sham animals (*n* = 11). This was however, not observed in either circular (d: Sham *n* = 7, 6-OHDA, *n* = 10), or longitudinal strips (e: Sham *n* = 10; 6-OHDA, *n* = 10) of the proximal colon. ∗ = Significant difference from Sham group; Mann–Whitney *U* test (f) *p* = 0.0133.

The spontaneous contraction of the distal colon showed no difference between the Sham and 6-OHDA animals in either of the tissues [Figure 6(a), 6(b), and 6(c)]. The average amplitude of the contractions showed also no difference between the groups (circular: 1.8 ± 0.2 mN vs. 2.0 ± 0.3 mN, unpaired *t*-test, *p* = 0.580; longitudinal: 2.7 ± 0.8 mN vs. 2.4 ± 0.3 mN, unpaired *t*-test, *p* = 0.713; segment: 2.9 ± 0.4 mN vs. 3.3 ± 0.5 mN, unpaired *t*-test, *p* = 0.482; for Sham and 6-OHDA, respectively).

In the proximal colon segments, significant increase in the frequency of spontaneous contraction was evident in the 6-OHDA (median: 35 contractions per 5 minutes) compared with Sham animals [median: 17 contractions per 5 minutes; Mann–Whitney *U* test, *p* = 0.0133; Figure 6(f)]. This change could not be seen in either circular or longitudinal tissue strips [Figures 6(d) and 6(e)]. The amplitudes of contractions showed no differences between the groups (circular: 2.2 ± 0.4 mN vs. 2.9 ± 0.9 mN, unpaired *t*-test, *p* = 0.525; longitudinal: 3.1 ± 0.9 mN vs. 3.6 ± 1.8 mN, unpaired *t*-test, *p* = 0.785; segment: 1.9 ± 0.4 mN vs. 1.6 ± 0.06 mN, unpaired *t*-test, *p* = 0.354; for Sham and 6-OHDA, respectively).

The distal ileum segments showed no difference in frequency of spontaneous contractions between the Sham and 6-OHDA groups [Figure 5(c)]. A non-significant difference was observed in the average contractile amplitude between the groups (1.6 ± 0.2 mN vs. 2.1 ± 0.2 mN, unpaired *t*-test, *p* = 0.0591; for Sham and 6-OHDA, respectively).

### 3.7. Contractile Responses in Healthy versus Sham-Operated Animals

When comparing the Healthy and the Sham-lesioned animals significant changes in both tissue strips and tissue segments were observed. The alterations were observed both in distal and proximal colon, but not distal ileum. Specifically in distal colon segments, significantly lower responses to high K^+^ Krebs were observed in the Sham group (0.52 ± 0.092 vs. 0.31 ± 0.034 mN mg^−1^, in Healthy (*n* = 7) and Sham (*n* = 17), respectively; unpaired *t*-test, *p* = 0.0141; Table 2). Similar lower responses were observed regarding EFS- and methacholine-induced contractility (two-way ANOVAs; EFS: group, *F*(1, 22) = 4.77, *p* = 0.0399; methacholine: group-concentration interaction, *F*(5, 110) = 3.61, *p* = 0.0046). The EFS-induced responses were also lower in the circular strips in this group (two-way ANOVA, group-concentration interaction, *F*(5, 70) = 3.63, *p* = 0.0057). Similar reduced responses to high K^+^ Krebs [Healthy (*n* = 7): 0.85 ± 0.015 mN mg^−1^vs. Sham (*n* = 7): 0.47 ± 0.075 mN mg^−1^; unpaired *t*-test, *p* = 0.0422; Table 2] and to methacholine-induced contractility (two-way ANOVAs group, *F*(1, 12) = 5.30, *p* = 0.0400) could also be observed in the circular strips of the proximal colon. In addition, decreased spontaneous contractions (median: 31 contractions per 5 minutes, in Healthy vs. 17 contractions per 5 minutes in the Sham animals; Mann–Whitney *U* test, *p* = 0.0050) were observed in segments.

## 4. Discussion

In the current study, we have shown the smooth muscle contractility of the distal colon and the proximal colon, but not in distal ileum, are significantly altered following 6-OHDA-induced central DA neurodegeneration at four weeks post-DA lesions. As has been previously reported by others [11, 13], we observed increased smooth muscle contractility of the distal colon following cholinergic stimulation in our segment preparations. However, we did not observe these changes in our circular or longitudinal strip preparations. One possible explanation for the discrepancies between our and others results' could be that we used full-thickness colon tissue strips, whereas previous mentioned studies used a protocol to remove the mucosal layers to isolate the smooth muscle only [30]. Furthermore, in the important previous study they used a cocktail of antagonists with and without tetrodotoxin to selectively investigate the cholinergic response only by the cholinergic agonist carbachol, whereas in the current study we investigated the response to the non-selective muscarinic agonist methacholine without any antagonists [11]. This indicates that effects of other neurotransmitters may be influencing the response observed in our full-thickness strip preparations. However, it is difficult to describe why only the full-thickness segments, but not the full-thickness strips, in this study showed alterations in contractility. The obvious difference is that the segments included more tissue, which may include more local ganglia with intact nerve fiber networks, but also intact connections between the circular and longitudinal muscle fibers and the mucosal layers. It is though important to stress that this is unexpected result in the current study, in particular when the circular or longitudinal tissue strip did not show any trend of increased contractility.

This study was not designed to comprehensively study the underlying mechanisms for changes in smooth muscle contractility in different sections of the gastrointestinal tract, but rather to understand differences in different types of tissue preparations. Based on the currently available literature, the observed increase in the cholinergic distal colon contractility is most likely due to a compensatory increase in muscarinic M3 receptor expression, caused by a decreased cholinergic transferase expression and subsequent decrease in release of acetylcholine in the 6-OHDA rat model [11, 13, 19, 22]. However, our data also revealed an increase in EFS-induced contractility in the segments (and no changes in the circular and longitudinal strips), which is in contrast to previous data showing significantly decreased contractions, in particular in the circular smooth muscle preparations, to electrical stimulation [11, 13, 14]. Interestingly, when Pellegrini et al. isolated the NK1-mediated tachykinergic response using EFS, longitudinal strips in fact showed a significantly increased contractility [14]. This could indicate that in our full-thickness tissue preparations, compensatory mechanisms originating from the mucosal layers may occur. The compensatory mechanisms likely also include transmitters, such as DA, serotonin, and noradrenaline. In addition, other factors that might be involved are vasoactive intestinal polypeptide (VIP), substance P, TH expression, and D_2_ receptor expression, which all have been shown to be altered in the distal colon in 6-OHDA rats [8, 9, 11, 12, 14, 18, 19, 22].

NO, apart from being involved in regulation of mucosal secretion, has a prominent role in gastrointestinal motility, acting as an inhibitory neurotransmitter causing relaxation of the smooth muscle tissue [31–33]. Furthermore, NO has been suggested to be directly involved in several gastrointestinal conditions including inflammatory bowel disease and constipation [34]. Considering this, we evaluated the role of NO in the observed changes in EFS and methacholine responses in the distal colon segments by using the NO synthase inhibitor L-NAME. The results showed significantly decreased methacholine-induced contractility in the 6-OHDA, and the absence of changes in Sham animals, following L-NAME administration in the segments. A similar tendency could also be observed regarding EFS-responses in the inverted segments. A significant increase in inducible NO synthase in macrophages in distal colon tissue has been seen in 6-OHDA rats, which may explain the involvement of NO in distal colon dysfunction after central DA degeneration [14]. Alterations in the inflammatory process and a disturbed mucosal barrier function in the colon, following central DA lesion, may further contribute to the observed changes in contractility [10, 13–15].

Alterations in contractility were observed in the proximal colon in the current study, mainly in the segments. We unexpectedly did not see any significant changes in EFS- or methacholine-induced responses, but rather changes attributed to significantly increased spontaneous contractility and maximal smooth muscle cell activation by high K^+^ Krebs solution. These alterations may be caused by previously reported changes in neuronal NO synthase expressing neurons, VIP expressing neurons, as well as TH expression and D_2_ receptors in the proximal colon of 6-OHDA-lesioned rats [8, 9, 22]. It is likely that a decreased expression of neuronal NO synthase could play a role in the increased spontaneous contractions [35]. In addition, alterations in dopaminergic signaling in the 6-OHDA-lesioned rats is also a plausible cause for the changes in the spontaneous contractions, whereas selective D_1_ antagonists have been shown to reduce peristalsis in the proximal colon [35]. However, it is important to note that the spontaneous contractions seen in isolated tissue is not equivalent to peristaltic waves, but rather an indication that these may be dysfunctional.

Our data showed no alterations in contractility in the distal ileum segments, indicating that this part of the gastrointestinal tract is not altered regarding contractile function by central DA lesion in the nigrostriatal pathway. However, similar to the proximal colon, histological and neurochemical evidence, including decreased neuronal NO synthase expressing neurons, increase in VIP-reactive neurons and decrease in mRNA expression of D_2_ receptors indicate that changes in the ileum in 6-OHDA-leisoned rats do occur [8, 9, 12]. In addition, altered spontaneous contractility of the ileum has also been reported in primate, marmoset, model of PD [36]. Together, this indicates that changes in contractile functionality in ileum need further investigation.

Lastly, regarding the aim to understand if Healthy animals could replace Sham-operated animals as control, the current data clearly showed that this are not possible. Several changes in muscle function, in particular the colon, were observed. Thus, the injection *per se* seems to be of importance. To specifically evaluate changes in gastrointestinal function as a result of DA loss in the nigrostriatal pathway by 6-OHDA, Sham-operated animals are therefore required as controls.

Future aspects from this study involves investigating the *in vitro* effects and possible role of other neurotransmitters, including DA, serotonin, and noradrenalin, using selective agonists and antagonists in the full-thickness segments of both proximal and distal colon in the 6-OHDA rat model. Once these functions and possible dysfunctions are established, *in vivo* studies need to be conducted to understand the direct involvement of the central nervous system. This, in turn, hopefully can lead to evaluation of specific drug targets in the gastrointestinal tract related to the characteristic central DA neurodegeneration seen in PD using, among other models, the 6-OHDA rat model of PD.

In conclusion, when studying smooth muscle dysfunction in rats in the 6-OHDA rat model, the choice of tissue preparation should be carefully considered when designing future studies and interpreting results. We suggest, based on the current data, which intact segments may be a better tissue preparation to study. This takes into account the complex interplay between circular and longitudinal contractions. Furthermore, apart from distal colon, alterations in smooth muscle contractility are also evident *in vitro* in the proximal colon, but not the distal ileum, following central DA loss caused by 6-OHDA. We believe that the current study sheds further light on the importance of study design and tissue preparations, but also on possible mechanisms of gastrointestinal dysfunction in PD.

## Figures and Tables

**Table 1:** Number of included tissue samples for electrical field stimulation (EFS) and methacholine in the Sham and 6-OHDA-lesioned and Healthy groups.

		EFS			Methacholine		
		Sham	6-OHDA	Healthy	Sham	6-OHDA	Healthy
Distal colon	Circular	*n* = 9	*n* = 10	*n* = 7	*n* = 11	*n* = 10	*n* = 9
	Longitudinal	*n* = 8	*n* = 9	*n* = 5	*n* = 10	*n* = 11	*n* = 10
	Segment	*n* = 17	*n* = 16	*n* = 7	*n* = 17	*n* = 16	*n* = 7
	Segment (inverted)	*n* = 6	*n* = 4	—	*n* = 6	*n* = 5	—
Proximal colon	Circular	*n* = 6	*n* = 7	*n* = 6	*n* = 7	*n* = 10	*n* = 7
	Longitudinal	*n* = 6	*n* = 7	*n* = 3	*n* = 10	*n* = 10	*n* = 9
	Segment	*n* = 8	*n* = 9	*n* = 5	*n* = 11	*n* = 11	*n* = 7
Distal ileum	Segment	*n* = 10	*n* = 9	*n* = 7	*n* = 11	*n* = 11	*n* = 7

**Table 2:** Tissue strip and segment weights (mg) and high K^+^ Krebs response (mN mg^−1^) in Sham-operated, 6-OHDA lesioned, and Healthy animals.

		Tissue weight (mg)					High K^+^ response per tissue weight (mN mg^−1^)				
		Sham	6-OHDA	*p*-Value*^a^*	Healthy	*p*-Value*^b^*	Sham	6-OHDA	*p*-Value^a^	Healthy	*p*-Value^b^
Distal colon	Circular	88.4 ± 10.2 (*n* = 11)	74.9 ± 11.5 (*n* = 10)	0.390	79.9 ± 8.3(*n* = 9)	0.542	0.61 ± 0.13 (*n* = 11)	0.57 ± 0.12 (*n* = 10)	0.839	0.70 ± 0.12 (*n* = 9)	0.618
	Longitudinal	70.1 ± 7.6 (*n* = 10)	85.2 ± 10.3 (*n* = 11)	0.261	76.2 ± 6.2 (*n* = 10)	0.541	0.35 ± 0.054 (*n* = 10)	0.26 ± 0.045 (*n* = 11)	0.209	0.33 ± 0.12 (*n* = 10)	0.837
	Segment	127.1 ± 8.0 (*n* = 17)	111.3 ± 9.8 (*n* = 16)	0.218	101.7 ± 5.1 (*n* = 7)	0.0636	0.31 ± 0.034 (*n* = 17)	0.51 ± 0.046 (*n* = 16)	***0.0010∗***	0.52 ± 0.092 (*n* = 7)	***0.0141∗***
	Segment (inverted)	187.0 ± 16.9 (*n* = 6)	137.3 ± 21.6 (*n* = 4)	0.104	—	—	0.013 ± 0.0025 (*n* = 6)	0.027 ± 0.0095 (*n* = 4)	0.122	—	**—**
Proximal colon	Circular	94.0 ± 10.5 (*n* = 7)	131.0 ± 15.3 (*n* = 10)	0.0896	90.5 ± 11.4 (*n* = 7)	0.827	0.47 ± 0.075 (*n* = 7)	0.70 ± 0.095 (*n* = 10)	0.105	0.85 ± 0.15 (*n* = 7)	***0.0422∗***
	Longitudinal	114.8 ± 13.3 (*n* = 10)	124.2 ± 12.0 (*n* = 10)	0.604	101.6 ± 17.4 (*n* = 9)	0.552	0.23 ± 0.033 (*n* = 10)	0.19 ± 0.030 (*n* = 10)	0.315	0.20 ± 0.044 (*n* = 9)	0.602
	Segment	159.6 ± 16.1 (*n* = 11)	138.5 ± 8.6 (*n* = 11)	0.260	166.1 ± 17.8 (*n* = 7)	0.796	0.30 ± 0.026 (*n* = 11)	0.43 ± 0.031 (*n* = 11)	***0.0028∗***	0.42 ± 0.071 (*n* = 7)	0.0614
Distal ileum	Segment	103.2 ± 8.7 (*n* = 11)	83.4 ± 5.9 (*n* = 11)	0.0734	91.3 ± 11.7 (*n* = 7)	0.418	0.19 ± 0.046 (*n* = 11)	0.18 ± 0.020 (*n* = 11)	0.842	0.22 ± 0.042 (*n* = 7)	0.686

^a^Unpaired *t*-test between Sham and 6-OHDA groups; ∗ and bold = significant differences between 6-OHDA and Sham groups.

^b^Unpaired *t*-test between Sham and Healthy animals; ∗ and bold = significant differences between Sham and Healthy groups.

## Data Availability

Data supporting this research article are available from the corresponding author or first author on reasonable request.

## Abbreviations

6-OHDA: 6-Hydroxydopamine
DA: Dopamine
EFS: Electrical field stimulation
NO: Nitric oxide
PFA: Paraformaldehyde
PD: Parkinson's disease
PB: Phosphate buffer
SEM: Standard error of the mean
TH: Tyrosine hydroxylase
VIP: vasoactive intestinal polypeptide

## Supplementary Materials

Supplementary Materials Supplementary Figure 1: Experimental design and timeline. In the beginning of the study the animals first received either an injection of saline (Sham, served as control), or the neurotoxin 6-hydroxydopmamine (6-OHDA) into the medial forebrain bundle of the nigrostriatal pathway. A third group of animals was left untreated, that is no brain surgery (Healthy). At 4 weeks after the stereotaxic surgery all animals (including Healthy animals) were deeply anesthetized and tissue samples of distal colon, proximal colon, and distal ileum were excised for *in vitro* organ bath analysis. The tissues were further prepared as either segments or strips, and mounted in the organ bath for muscle contractility evaluation. All tissues first received stimulation by high K^+^ Krebs followed by electrical field stimulation (EFS; 1–40 Hz) and direct cholinergic receptor stimulation with the muscarinic agonist methacholine (10^−8^–10^−3^ M). All tissue were also examined for EFS- and methacholine-induced contractility in the presence of the cholinergic antagonist atropine (10^−6^ M). In a subset of the distal colon segments, including inverted segments, the tissues were evaluated in the presence of the nitric oxide synthase inhibitor L-NAME. In addition, the brains were transcardial fixed by paraformaldehyde (PFA) perfusion and further sectioned and stained immunohistochemically for tyrosine hydroxylase (TH) to confirm the dopamine lesion (6-OHDA), or the absence of any lesion (Sham).
