# Supplementary figures and images for: 6-OHDA-Induced Changes in Colonic Segment Contractility in the Rat Model of Parkinson's Disease
Source: Gastroenterol Res Pract. 2023 Jan 27;2023:9090524. doi: 10.1155/2023/9090524 (PMC9897937; doi:10.1155/2023/9090524)

## Experimental design and time-line

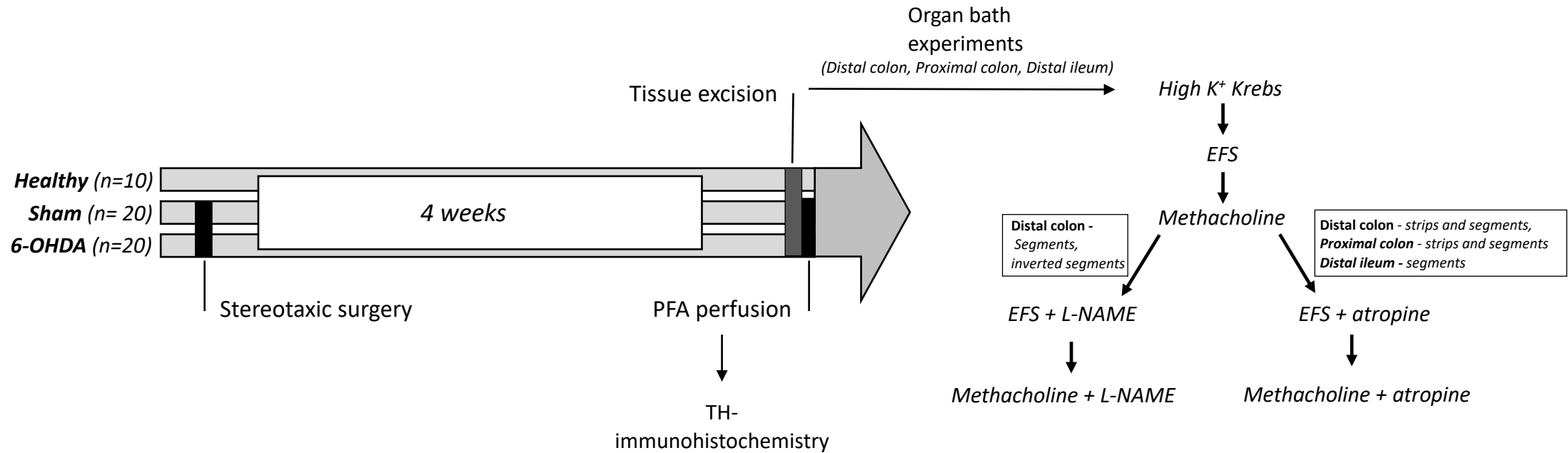

Supplement: Supplementary Materials — Supplementary Figure 1: Experimental design and timeline. In the beginning of the study the animals first received either an injection of saline (Sham, served as control), or the neurotoxin 6-hydroxydopmamine (6-OHDA) into the medial forebrain bundle of the nigrostriatal pathway. A third group of animals was left untreated, that is no brain surgery (Healthy). At 4 weeks after the stereotaxic surgery all animals (including Healthy animals) were deeply anesthetized and tissue samples of distal colon, proximal colon, and distal ileum were excised for in vitro organ bath analysis. The tissues were further prepared as either segments or strips, and mounted in the organ bath for muscle contractility evaluation. All tissues first received stimulation by high K+ Krebs followed by electrical field stimulation (EFS; 1–40 Hz) and direct cholinergic receptor stimulation with the muscarinic agonist methacholine (10−8–10−3 M). All tissue were also examined for EFS- and methacholine-induced contractility in the presence of the cholinergic antagonist atropine (10−6 M). In a subset of the distal colon segments, including inverted segments, the tissues were evaluated in the presence of the nitric oxide synthase inhibitor L-NAME. In addition, the brains were transcardial fixed by paraformaldehyde (PFA) perfusion and further sectioned and stained immunohistochemically for tyrosine hydroxylase (TH) to confirm the dopamine lesion (6-OHDA), or the absence of any lesion (Sham). [file 9090524.f1.pdf]
